# Tailoring far-infrared surface plasmon polaritons of a single-layer graphene using plasmon-phonon hybridization in graphene-LiF heterostructures

**DOI:** 10.1038/s41598-018-31049-6

**Published:** 2018-09-04

**Authors:** Hodjat Hajian, Andriy E. Serebryannikov, Amir Ghobadi, Yigit Demirag, Bayram Butun, Guy A. E. Vandenbosch, Ekmel Ozbay

**Affiliations:** 1 Nanotechnology Research Center, Bilkent University, 06800 Ankara, Turkey; 2 ESAT-TELEMIC, Katholieke Universiteit Leuven, B-3000 Leuven, Belgium; 3 Faculty of Physics, Adam Mickiewicz University, 61-614 Poznan, Poland; 4 Department of Electrical and Electronics Engineering, Bilkent University, 06800 Ankara, Turkey; 5 Department of Physics and UNAM-Institute of Materials Science and Nanotechnology, Bilkent University, 06800 Ankara, Turkey

## Abstract

Being one-atom thick and tunable simultaneously, graphene plays the revolutionizing role in many areas. The focus of this paper is to investigate the modal characteristics of surface waves in structures with graphene in the far-infrared (far-IR) region. We discuss the effects exerted by substrate permittivity on propagation and localization characteristics of surface-plasmon-polaritons (SPPs) in single-layer graphene and theoretically investigate characteristics of the hybridized surface-phonon-plasmon-polaritons (SPPPs) in graphene/LiF/glass heterostructures. First, it is shown how high permittivity of substrate may improve characteristics of graphene SPPs. Next, the possibility of optimization for surface-phonon-polaritons (SPhPs) in waveguides based on LiF, a polar dielectric with a wide polaritonic gap (Reststrahlen band) and a wide range of permittivity variation, is demonstrated. Combining graphene and LiF in one heterostructure allows to keep the advantages of both, yielding tunable hybridized SPPPs which can be either forwardly or backwardly propagating. Owing to high permittivity of LiF below the gap, an almost 3.2-fold enhancement in the figure of merit (*FoM*), ratio of normalized propagation length to localization length of the modes, can be obtained for SPPPs at 5–9 THz, as compared with SPPs of graphene on conventional glass substrate. The enhancement is efficiently tunable by varying the chemical potential of graphene. SPPPs with characteristics which strongly differ inside and around the polaritonic gap are found.

## Introduction

After the rise of graphene, the two-dimensional periodic array of carbon atoms arranged in a honeycomb lattice^1,2^, a great deal of attention has been attracted to its potential applications in optoelectronics^3^ and plasmonics^4^. As a plasmonic material, graphene offers such intriguing properties as crystalline stability^5^, large optical nonlinearities^6–8^ and extremely high electromagnetic field concentration^9^. Recent demonstrations of surface plasmon polariton (SPP) excitation in graphene using near-field scattering of infrared light^10^, with all-optical mechanisms^11^, and by structure patterning^12,13^ have received intense interests. The surface conductivity of graphene (*σ*_*g*_) can be effectively modulated via tuning of chemical potential (*μ*) through chemical doping or electrostatic/magnetostatic gating^1,2^. When *Im*(*σ*_*g*_) > 0, graphene behaves like a very thin metal layer capable of supporting transverse-magnetic (TM) guided plasmonic modes^14–24^. Tunability of its plasmon resonance through the variation of *μ* together with a relatively large propagation length (*PL*) and a small localization length (*LL*) of SPPs in the mid-infrared (IR)^20^, far-IR and terahertz (THz) ranges^25,26^ are the key advantages of the graphene SPPs over those supported by noble metals like silver and gold^27^. It has been experimentally demonstrated that the plasmonic modes in graphene can be confined in extremely small volumes. The modal confinement can be ∝10^7^ times smaller than free space wavelengths^20,28^. To compare, surface plasmons supported by normal metals typically display mode volumes that are ∝10^3^ times smaller than free space with similar damping^27^. Therefore, graphene plasmons can couple stronger to electronic and vibronic excitations of their local environment than normal metal plasmons.

Alongside the noble metals and graphene, polar dielectrics also offer simultaneous sub-diffractional confinement, low optical losses, and operation in the mid-IR to THz spectral ranges through the stimulation of surface phonon polariton (SPhP) modes^29^. SPhPs originate from the interaction of optical phonons with long-wavelength incident fields, creating a surface excitation mediated by the atomic vibrations. Depending on the type of the polar material, SPhPs can be recognized in a wide frequency range from mid-IR (hexagonal boron nitride (hBN)^30,31^ and SiC^32,33^) to far-IR (LiF^34,35^, GaAs^36–39^, InP and CaF_2_^29^). Such SPhP modes can be stimulated between the longitudinal optical (LO) and transverse optical (TO) phonon frequencies. This spectral range is referred as the Reststrahlen (RS) band or polaritonic gap. According to the above-mentioned eye-catching characteristics of graphene SPPs and the SPhPs of polar dielectrics in the THz and IR regions, heterostructures combining advantages of graphene and polar dielectrics can yield unique and useful optical responses. Recent experimental and theoretical studies on graphene on SiO_2_^28,40,41^ and SiC^42–44^ substrates have shown that the graphene dispersion relation in the mid-IR range can be significantly modified due to the substrate phonons with extra modes arising due to plasmon-phonon coupling. This coupling has been considered as surface-phonon-plasmon-polaritons, or SPPPs^28^. However, in those studies it was not tested whether the graphene plasmons were coupled to a large volume of phonons spread throughout the dielectric environment, or only to the phonons in the immediate vicinity of the graphene sheet. The recent experiments aimed to clarify the coupling between graphene plasmons and phonons of thin layers of PMMA show that the PMMA phonon spectral signature can be enhanced through graphene plasmon coupling, at least for the PMMA layers as thin as 8 *nm*^45^. Hexagonal boron nitride (hBN) is another polaritonic material that operates in the mid-IR and can be exfoliated as mono-/multi-layer atomically thin films^46,47^ or thick films (>50 *nm*)^30,31^. In^46,47^, by fabricating patterned graphene/monolayer-hBN and graphene/triple-layer-hBN structures, it was proved that graphene plasmons can couple to optical excitations occupying an atomically thin slice of volume near the graphene. In this case, two clearly separated hybridized SPPP modes can be formed. At the same time, the three-dimensional specimens of this layered anisotropic material are capable of supporting hyperbolic phonon polaritons^30,31^. Combining graphene with hBN films in multilayer structures, one can tune the hyperbolic dispersion of hBN phonons^48–51^. Moreover, the coupling of graphene plasmons and hyperbolic phonons in multilayer graphene-hBN metamaterials leads to the appearance of hybrid plasmon-phonon-polariton bands that makes this structure beneficial for waveguiding, negative refraction, and hyperlensing^52^.

While plasmon-phonon coupling in graphene-polar structures has been studied in^28,40–52^ in the mid-IR region, similar effects are expected to be realizable but have not yet been studied in the far-IR range, in which graphene possess numerous potential applications^4,34^. However, it remains unclear whether the improvement of the resulting characteristics for the mid-IR by the use of hNB can be replicated in the far-IR region by using polar dielectrics, for which phonon-photon interaction occurs at lower frequencies. More general questions are (i) whether SPPPs obtained due to a polar dielectric substrate, which is highly dispersive, may have advantages over SPPs obtained for a conventional dielectric substrate, and (ii) which properties of polar dielectric are required to obtain better characteristics of SPPPs at the far-IR. One more important question is whether the properties of the SPPPs in case of polar-dielectric substrate can be predicted based on the characteristics of the SPPs in case of conventional dielectric substrate. Hence, possible effects of variations in permittivity of substrate should be clarified starting from the case of dispersion-free dielectric substrate.

In this paper, we theoretically investigate the far-IR propagation and localization characteristics of SPPs of a single-layer graphene on dispersion-free dielectric substrates and SPPPs supported by the single-layer graphene on a thin film of LiF, a polar dielectric. First, we consider graphene SPPs for four different substrates and demonstrate importance of the choice of substrate permittivity and chemical potential of graphene on the resulting characteristics. In particular, we will show that high-permittivity substrates allow one to significantly improve figure of merit (*FoM*) at *f* < 20 *THz*. Since polar dielectrics typically show strong variations in permittivity inside and around their polaritonic gap (RS band), they are good candidates to be high-permittivity substrates for single-layer graphene at the far-IR. On the other hand, they may themselves support SPhPs. Thus, we next investigate SPhPs supported by thin films of LiF. The capability of a 10 *nm* thick LiF waveguide on glass substrate in support of the highly confined and long-range phononic modes at 10–18 *THz* will be discussed in detail. Then, we combine single-layer graphene and LiF film in one structure in order to keep the advantages of both tunability and wide-range variations of permittivity, and study the propagation and localization characteristics of SPPPs of this heterostructure. It will be shown that the high-permittivity region of LiF can positively affect the SPPPs characteristics below the polaritonic gap, in coincidence with our results for SPPs, obtained in the case of dispersion-free lossless substrates. Moreover, it is found that transition from forward to backward SPPPs may occur inside and just below the polaritonic gap. To the best of our knowledge, the results presented in this paper is the first attempt to systematically study the effects of dispersion-free and dispersive polar substrates on graphene surface plasmons characteristics at the far-IR region.

## Results and Discussion

### Effects of dielectric substrate on characteristics of surface plasmon polaritons of a single-layer graphene

The use of a dielectric substrate is unavoidable for feasibility of the design and often enables advanced physical regimes and operating modes. Various electromagnetic phenomena can be efficiently controlled by a proper choice of substrate^53–57^. Effects exerted by substrate can often be understood through the prism of scaling by assuming that the same characteristics can be obtained at higher or lower frequencies, if the structure is properly modified. The classical scaling rule of resonance frequencies, $f\propto {\varepsilon }_{d}^{-1/2}$ (*ε*_*d*_ is permittivity of the dielectric filling a cavity), is known for lossless cavities^58^. Prediction of scaling capability of an open resonance structure is a challenging task, since there is no exact boundary of the region occupied by the resonance field. A proportional change of geometrical (and material) parameters enables scaling of an existing transmittive/reflective structure with respect to frequency, provided that dispersion and losses in the used materials are relatively weak^59^. Besides, partial scaling is possible by varying permittivity of substrate and other dielectric components, while all geometrical sizes are kept fixed^57,60^. Alongside the cavities and transmittive/reflective configurations, effect of dielectric substrate on propagation of plasmons in silver nanowires shows up as interesting characteristics^53^. Intuitively, increase of permittivity leads to downscaling (for fixed frequency) or redshift (for fixed geometry). However, the quantifying of these effects is impossible without a detailed numerical study. Therefore, in order to investigate influence of substrate on SPPs of a single-layer graphene, in this section we numerically study the basic effects of variation of permittivity of a lossless, dispersion-free dielectric substrate, *ε*_*d*_, on the SPPs characteristics, i.e., propagation length, localization length, and figure of merit.

**Figure 1:**
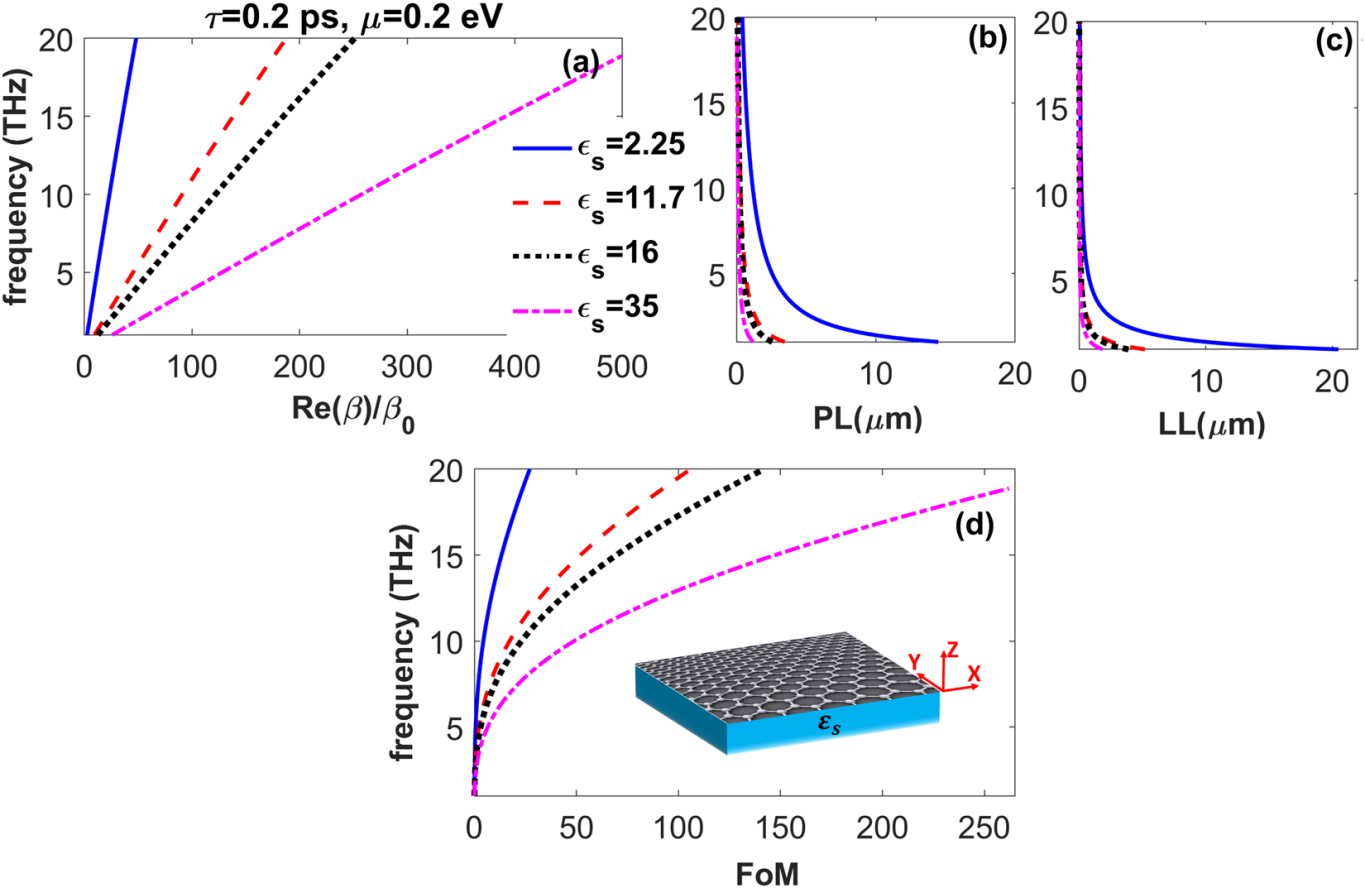
(**a**) Dispersion, (**b**) propagation length, (**c**) localization length, and (**d**) figure of merit of the SPPs supported by a single-layer graphene on various dielectric substrates at *μ* = 0.2 *eV*; *ε*_*s*_ = 2.25 (solid blue line), 11.7 (dotted-dashed red line), 16 (dotted black line), and 35 (dashed pink line). The schematic of the studied structure is depicted in panel (d), inset.

The schematic of the studied structure is shown in Fig. 1(d), inset. We consider a sheet of graphene placed at *z* = 0 on a semi-infinite lossless substrate with permittivity *ε*_*s*_. Taking *y* component of the magnetic field as

1 $${H}_{y}(z)=\{\begin{array}{cc}a{e}^{-{q}_{a}(z)} & z > 0\\ s{e}^{{q}_{s}(z)} & z < 0\end{array}\},$$

and applying the boundary conditions for TM polarization^14,61^, we arrive at the following well-known dispersion relation of graphene SPPs^14–22,25^:

2 $${\varepsilon }_{a}/{q}_{a}+{\varepsilon }_{s}/{q}_{s}=\alpha $$

where ${q}_{i}=\sqrt{{\beta }^{2}-{\varepsilon }_{i}{\beta }_{0}^{2}}$ (*i* = *a*, *s*), *β* = *k*_*x*_, *β*_0_ = *ω*/*c*, and *α* = *σ*_*g*_/*iωε*_0_. The optical conductivity of graphene (${\sigma }_{g}={\sigma }_{g}^{{intra}}+{\sigma }_{g}^{{inter}}$) can be presented as^14^

3a $${\sigma }_{g}^{{intra}}=\frac{{e}^{2}}{4\hslash }\frac{i}{2\pi }\{\frac{16{k}_{B}T}{\hslash {\rm{\Omega }}}\,\mathrm{ln}(2\,{\cosh }(\frac{\mu }{2{k}_{B}T}))\},$$

3b $${\sigma }_{g}^{{inter}}=\frac{{e}^{2}}{4\hslash }\{\frac{1}{2}+\frac{1}{\pi }\,\arctan (\frac{\hslash {\rm{\Omega }}-2\mu }{2{k}_{B}T})-\frac{i}{2\pi }\,\mathrm{ln}\,\frac{{(\hslash {\rm{\Omega }}+2\mu )}^{2}}{{(\hslash {\rm{\Omega }}-2\mu )}^{2}+{(2{k}_{B}T)}^{2}}\},$$

with Ω = *ω* + *iτ*^−1^, *μ* is chemical potential of graphene, *e* is the electron charge, *k*_*B*_ is the Boltzmann constant, ℏ is the Plank constant over 2*π*, and *c* is the speed of light in vacuum. Here, we take the electron relaxation time and temperature as *τ* = 0.2 *ps* (otherwise stated) and *T* = 300 *K*, respectively. Moreover, considering *β* = *β*′ + i*β*″, we define wavelength, propagation length and localization length of the guided modes as *λ*_*sp*_ = 2*π*/*β*′, *PL* = 1/2*β*″ and *LL* = 1/2*Re*(*q*_*a*_ + *q*_*s*_), respectively, and figure of merit as *FoM* = (*PL*/*λ*_*sp*_)/*LL*(*μm*). For the sake of simplicity, we use the notation *λ*_*sp*_ for surface waves of all types studied here, i.e., SPPs, SPhPs and SPPPs.

First, we examine the effect of different, dispersion-free lossless substrates on the characteristics of graphene SPPs for *μ* = 0.2 *eV*. The considered substrates include glass (*ε*_*s*_ = 2.25), Si (*ε*_*s*_ = 11.7), Ge (*ε*_*s*_ = 16), and a material with *ε*_*s*_ = 35^60^. It is seen in Fig. 1(a) that by increasing *ε*_*s*_, graphene SPPs are supported for larger values of the wavenumber at fixed frequencies, and, consequently can propagate with smaller *λ*_*sp*_ and group velocity, *v*_*g*_. The scaling is applicable to the results in Fig. 1(a), i.e., an ${\varepsilon }_{s}^{-\mathrm{1/2}}$-like fitting can be introduced similarly to^57^. Note that such large wavenumbers like those in Fig. 1(a) at *ε*_*s*_ = 35 can also be achieved using patterned graphene on conventional substrate^12^. Figure 1(b,c) show that the increase in *ε*_*s*_ leads to decrease in *PL* and *LL*. However, as observed in Fig. 1(d), *FoM* of the plasmonic guided modes becomes considerably larger at *f* > 5 *THz*, that originates from decrease in *λ*_*sp*_ and *LL* of the graphene SPPs. Thus, increase of *FoM* is coherent here with decrease of *v*_*g*_.

It is known that one of the most impactful properties of the SPPs supported by graphene is their tunability by varying *μ*. In Fig. S1 (see Supplementary Information) by taking *ε* = 35, we examine the SPP characteristics for different values of *μ*. We found that the effect of increase of *μ* is similar to the effect of decrease of *ε*_*s*_ for *λ*_*sp*_, but the same cannot be said regarding *FoM*, for which an optimal value of *ε*_*s*_ can exist. Moreover, for a richer insight into the localization of the graphene SPPs, field profiles are also presented in Fig. S2 at four different frequencies.

### SPhPs of thin LiF films

According to the results discussed in Sect. 2, the larger value of *ε*_*s*_, the larger values of *FoM* are obtainable for the graphene SPPs. High-permittivity dispersion-free materials are not accessible for the far-IR. Hence, the choice of polar dielectrics like LiF^34,35^, NaCl^34^, and GaAs^36–38^ is natural. These materials are known to show a polaritonic gap (RS band), which appears due to phonon-photon interaction. Desired high permittivity values result from this interaction. Graphene SPPs can be hybridized at the far-IR with phonons in heterostructures composed of graphene and polar materials. From a fabrication point of view, those materials should be used as a buffer layer between a single-layer graphene and a thicker low-loss dielectric substrate. Therefore, a study of the waveguide structures comprising thin films of a polar dielectric on low-loss dielectric substrate is required prior to combining with graphene. In this section, we scrutinize propagation and localization characteristics of SPhPs supported by thin films of LiF on different lossless substrates, within 1 *THz* to 20 *THz*. LiF is particularly appropriate due to the wide RS band and a wide range of permittivity variation inside and around this band^34,35^.

**Figure 2:**
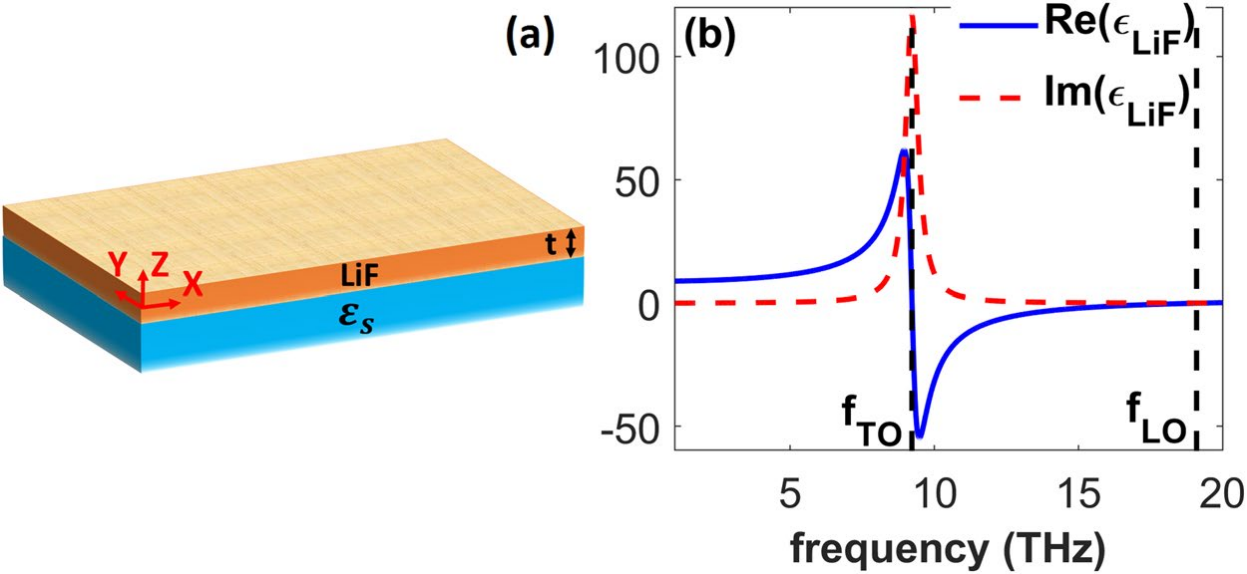
(**a**) Schematic of the structure comprising a thin film of LiF with thickness *t* that is centered at *z* = 0 and placed on a lossless substrate. (**b**) Real (solid blue line) and imaginary (dashed red line) parts of *ε*_*LiF*_ within 1–20 *THz*; vertical dashed lines correspond to *f*_*TO*_ = 9.22 *THz* and *f*_*LO*_ = 19.1 *THz* of LiF, being the boundaries of the polaritonic gap (RS band).

Figure 2(a) schematically shows a LiF film on the top of a substrate with *ε*_*s*_. The considered LiF has thickness *t* and permittivity given by^34^

4 $${\varepsilon }_{LiF}={\varepsilon }_{\infty }(1-\frac{{\omega }_{LO}^{2}-{\omega }_{TO}^{2}}{{\omega }^{2}-{\omega }_{TO}^{2}-i\gamma \omega }),$$

where *ε*_∞_ = 2.027, *ω*_*TO*_ = 2*πf*_*TO*_, *ω*_*LO*_ = 2*πf*_*LO*_, *f*_*TO*_ = 9.22 *THz*, *f*_*LO*_ = 19.1 *THz* and *γ* = 2*π* × 0.527 *THz*. Here, *f*_*TO*_, *f*_*LO*_ and *γ* are, respectively, transverse optical frequency, longitudinal optical frequency and damping factor. Real and imaginary parts of *ε*_*LiF*_ are shown in Fig. 2(b). It is obvious from Fig. 2(b) that *Re*(*ε*_*LiF*_) takes considerably large values at frequencies slightly smaller than *f*_*TO*_. A thin layer of LiF that has thickness *t* and is bounded by air half-space (*ε*_*a*_ = 1) on the one side and by a typical dielectric substrate (*ε*_*s*_) on the other side can support SPhPs. Taking *H*_*y*_ as

5 $${H}_{y}(z)=\{\begin{array}{cc}a{e}^{-{q}_{a}(z-t/2)} & z > t/2\\ {l}_{1}{e}^{-{q}_{LiF}(z)}+{l}_{2}{e}^{{q}_{LiF}(z)} & -t/2\le z\le t/2\\ s{e}^{{q}_{s}(z+t/2)} & z < -\,t/2\end{array}\},$$

and then applying the TM-case boundary conditions, we arrive at the following dispersion relation of the SPhPs that are supported by the LiF layer on the dielectric substrate:

6 $${\tanh }({q}_{LiF}t)=-\,\frac{{{\rm{\Gamma }}}_{a}+{{\rm{\Gamma }}}_{s}}{1+{{\rm{\Gamma }}}_{a}{{\rm{\Gamma }}}_{s}},$$

where Γ_*a*_ = *q*_*LiF*_*ε*_*a*_/*q*_*a*_*ε*_*LiF*_, Γ_*s*_ = *q*_*LiF*_*ε*_*s*_/*q*_*s*_*ε*_*LiF*_, and ${q}_{LiF}=\sqrt{{\beta }^{2}-\varepsilon {\beta }_{0}^{2}}$.

**Figure 3:**
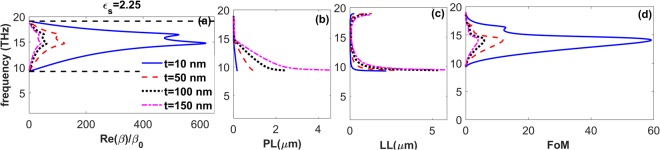
(**a**) Dispersion, (**b**) propagation length, (**c**) localization length, and (**d**) figure of merit of the SPhPs supported by air/LiF waveguides of thickness *t* on a glass substrate, respectively; *t* = 10 *nm* (solid blue line), *t* = 50 *nm* (dashed red line), *t* = 100 *nm* (dotted black line), and *t* = 150 *nm* (dashed-dotted pink line). Dashed black lines in panel (a) show the edges of the polaritonic gap of LiF.

First, we investigate characteristics of the SPhPs for different thicknesses of the LiF layer placed on a semi-infinite glass substrate (*ε*_*s*_ = 2.25). The obtained results are presented in Fig. 3. It is seen in Fig. 3(a) that SPhPs are supported in the polaritonic gap (RS band) of LiF, in which *Re*(*ε*_*LiF*_) < 0. At *t* = 10 *nm*, they can propagate with large wavenumbers, *Re*(*β*)/*β*_0_ < 640, i.e., with small phonon wavelengths (*λ*_*sp*_). By increasing *t*, SPhPs with larger *λ*_*sp*_ can be supported, as occurs for *t* = 150 *nm* when *Re*(*β*)/*β*_0_ < 50. Figure 3(b) illustrates that the larger thickness of the phononic waveguides, the larger *PL* is achievable for the SPhPs. In particular, the maximum *PL* is obtained at *t* = 150 *nm*, while the minimum value of *PL* belongs to the case of *t* = 10 *nm*. More investigations show that taking *t* = 200 *nm*, even larger values of *PL* can be obtained. In turn, the results presented in Fig. 3(c) indicate that the thinner the phononic waveguide is, the higher confinement of the SPPs can be obtained. In other words, the minimum *LL* is achieved at *t* = 10 *nm*. The *FoM* is presented in Fig. 3(d). The maximum values of the *FoM* are obtained at *t* = 10 *nm*. By increasing *t*, one reduces the *FoM* value. Consequently, as far as supporting the phononic guided modes with lower losses (larger values of *PL*/*λ*_*sp*_) and stronger localization (smaller values of *LL*) within 10–18 *THz* are concerned, the smaller thickness of the phononic waveguide can be chosen. One more point that should be highlighted here is the support of phononic guided modes with *negative slope* of dispersion. These modes have been considered here as backward waves, being similar to the ones supported by the left-handed metamaterials^62,63^, and by metal-insulator-metal^64,65^ and insulator-metal-insulator^66^ structures. The total power $1/2{Re}[\int \,{S}_{x}{d}_{z}]$ of the guided modes with backward propagation is negative (*S*_*x*_ is x-component of the Poynting vector, *S*).

Similar to the the previous section, we have also investigated the effect of *ε*_*s*_ on the modal characteristics of the LiF waveguide. The results are presented in Fig. S3 (see Supporting Information). To get more insight into the SPhPs characteristics, their *FoM* for different values of *t* (*t* = 50 *nm*, 100 *nm*, 150 *nm* and 200 *nm*) and *ε*_*s*_ are illustrated in Fig. S4. As a complementary discussion, in Fig. S5, we also investigated typical mode profiles of the LiF phononic waveguide for *t* = 10 *nm* and *t* = 100 *nm*. As comprehensively discussed in the the Supporting Information, we found that the SPhPs of the 10 *nm*-thick LiF waveguide are as confined as the graphene SPPs in 10–18 *THz*.

### SPPPs of graphene-LiF waveguide on glass substrate

**Figure 4:**
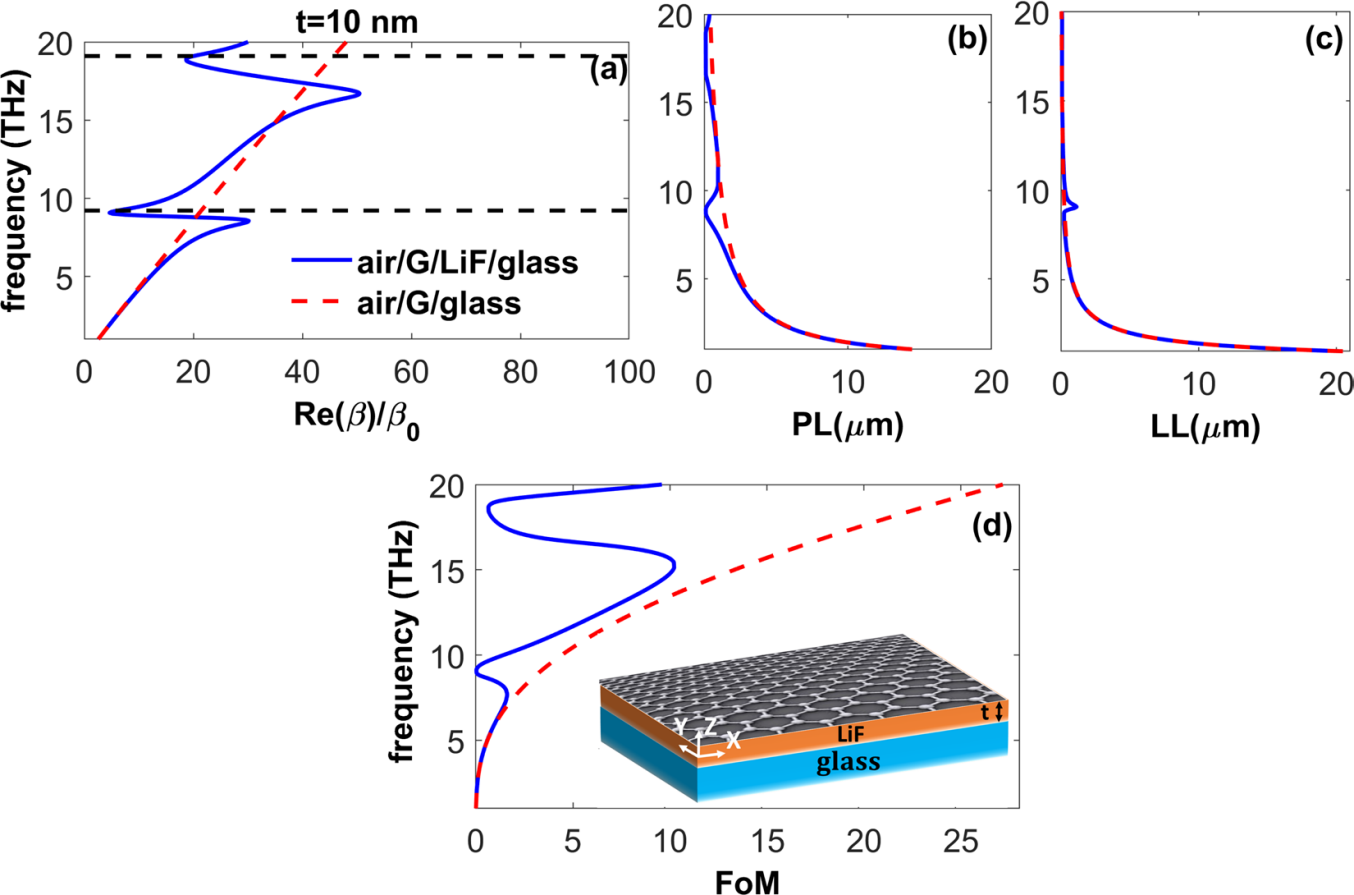
(**a**) Dispersion, (**b**) propagation length, (**c**) localization length, and (**d**) figure of merit of the SPPPs supported by air/graphene/LiF/glass waveguide with *t* = 10 *nm* (solid blue lines); the schematic of the studied structure is presented in panel (d), inset. For the sake of comparison, results are also presented for the SPPs of the graphene/glass structure (dashed red lines). Here, *μ* = 0.2 *eV* and the dashed black horizontal lines in panel (a) show the edges of the polaritonic gap of LiF.

To keep the advantages of tunability of graphene and a wide permittivity range of LiF, we combine them in one hybrid structure so that graphene SPPs could be hybridized with SPhPs. As schematically illustrated in Fig. 4(d), inset, the structure in Fig. 2 is modified now by placing a single layer of graphene on top of the LiF film. Substituting the following equation^25,67^

7 $${{\rm{\Gamma }}}_{a}=\frac{{q}_{LiF}}{{\varepsilon }_{LiF}}(\frac{{\varepsilon }_{a}}{{q}_{a}}-\alpha )$$

into Eq. (6), we obtain the dispersion relation of the coupled plasmonic-phononic modes of this system, which are labeled as surface-phonon-plasmon-polaritons (SPPPs)^28^. According to the results presented in the first part of the Results and Discussion section, we found that the SPPs of a single layer of graphene possess larger values of *FoM* for larger values of *ε*_*s*_. Hence, due to the large values of *Re*(*ε*_*LiF*_) at the frequencies below but close to *f*_*TO*_, unique modal characteristics are expected to appear. In this section, our consideration is restricted to the case when glass is the substrate of the graphene-LiF heterostructure. As discussed in the previous part, the 10 *nm*-thick LiF waveguide on glass substrate shows the largest *FoM* values due to the support of low-loss SPhPs with strong confinement [see Fig. S5(a,b)]. Therefore, in Fig. 4, we present the characteristics of the SPPPs of the waveguide structure at *t* = 10 *nm*. In Fig. 4(a), one can see that SPPPs dispersion is noticeably modified as compared with SPhPs dispersion of the 10 nm-thick LiF waveguide in Fig. 3(a) and dispersion of the graphene SPPs. Due to the plasmon-phonon coupling, first, the SPPPs can be supported at considerably smaller wavenumbers and, second, backward SPPPs can propagate inside the waveguide for two frequency ranges: 8.63 *THz* < *f* < *f*_*TO*_ and 16.83 *THz* < *f* < 18.68 *THz*. These two features cannot be observed in the air/graphene/glass and air/LiF/glass waveguides separately, i.e., without combining them in one structure. Figure 4(b,c) indicate that the combination of *highly confined* SPhPs of LiF with the localized graphene SPPs leads to a giant increase in the losses of the guided modes, so a considerable decrease/increase in the *PL*/*LL* values of the SPPPs occurs as compared to the graphene SPPs. As illustrated in Fig. 4(d), the 10 nm-thick graphene-LiF waveguide supports SPPPs with much smaller *FoM* values than the graphene SPPs.

**Figure 5:**
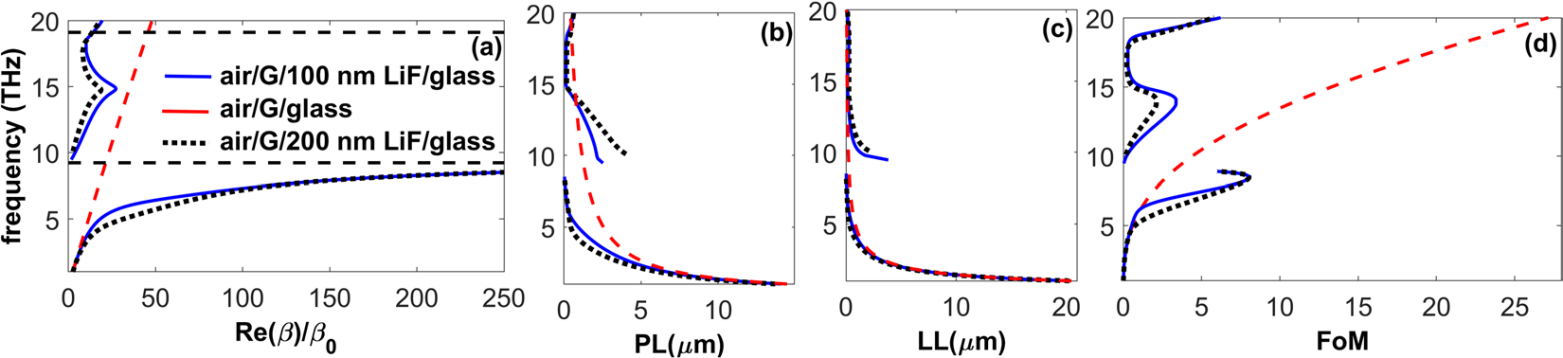
(**a**) Dispersion, (**b**) propagation length, (**c**) localization length, and (**d**) figure of merit of the SPPPs supported by the air/graphene/LiF/glass waveguide for *t* = 100 *nm* (solid blue lines), *t* = 200 *nm* (dotted black lines) at *μ* = 0.2 *eV*. Results for SPPs of the graphene/glass structure are presented for comparison (dashed red lines). The edges of the polaritonic gap of LiF are shown by dashed black line.

From the results in Figs 3 and S4 it was found that by increasing the thickness of the polaritonic core material in the LiF waveguides, *FoM* of the SPhPs is decreased due to the confinement reduction. Therefore, it is worth to investigate the specifics of coupling between the highly confined graphene SPPs and the lowly confined SPhPs of air/LiF/glass system for two larger values of the thickness of LiF; i.e. *t* = 100 *nm* and *t* = 200 *nm*. As it was expected from the results shown in Fig. 4(a), the modes with negative slope of dispersion are supported at *f* > 14.86 *THz* by the air/graphene/LiF/glass waveguides for both *t* = 100 *nm* and *t* = 200 *nm*, see Fig. 5(a). Even more interestingly, Fig. 5(a) demonstrates that, in contrast with the case of *t* = 10 *nm*, for the larger *t*, the backward modes that occur for 8.63 *THz* < *f* < *f*_*TO*_ are turned to be forwardly propagating SPPPs with very small *v*_*g*_ and *λ*_*sp*_ in the vicinity of 8.63 *THz*. As is seen from Fig. 5(b,c), despite the SPPPs with *f* < *f*_*TO*_ possess smaller values of *PL* than those of graphene SPPs, they are more strongly confined; i.e., they also possess smaller values of *LL*. This means that the hybridization of the highly confined graphene SPPs with the lowly confined SPhPs of air/LiF/glass structure leads to the extremely confined SPPPs at frequencies below *f*_*TO*_, where *Re*(*ε*_*LiF*_) is large. This decrease in the localization length of the modes yields a considerable rise in the *FoM* of the SPPPs at 5.2 *THz* < *f* < *f*_*TO*_, compared to graphene SPPs illustrated in Fig. 5(d). Thus, an almost 3.2-fold enhancement of *FoM* is achieved around 8.5 *THz*. Therefore, hybridization of graphene SPPs with the lowly confined SPhPs leads to the appearance of SPPPs with larger *FoM* than a single layer of graphene. It should be noted that since LiF is a lossy material, its presence reduces *PL*, however decreases *LL* as well for the cases of *t* = 100 *nm* and 200 *nm*. Similar to first part of the Result and Discussion section, increase of *FoM* is associated in this regime with the slowing effect of the substrate. In the case we are only interested in supporting SPPPs with larger *PL* than for graphene SPPs, air/graphene/LiF/glass waveguides with *t* = 100 *nm* and *t* = 200 *nm* are the appropriate designs for the range of 9.45 *THz* < *f* < 13.77 *THz*. In line with the said above, support of SPPPs with significantly different propagation and localization characteristics within three neighboring frequency bands (i.e., below, inside, and above the polaritonic gap) makes the suggested air/graphene/LiF/glass heterostructure a distinguishable candidate for multifunctional applications including, for instance, waveguiding, sensing, and absorption purposes.

**Figure 6:**
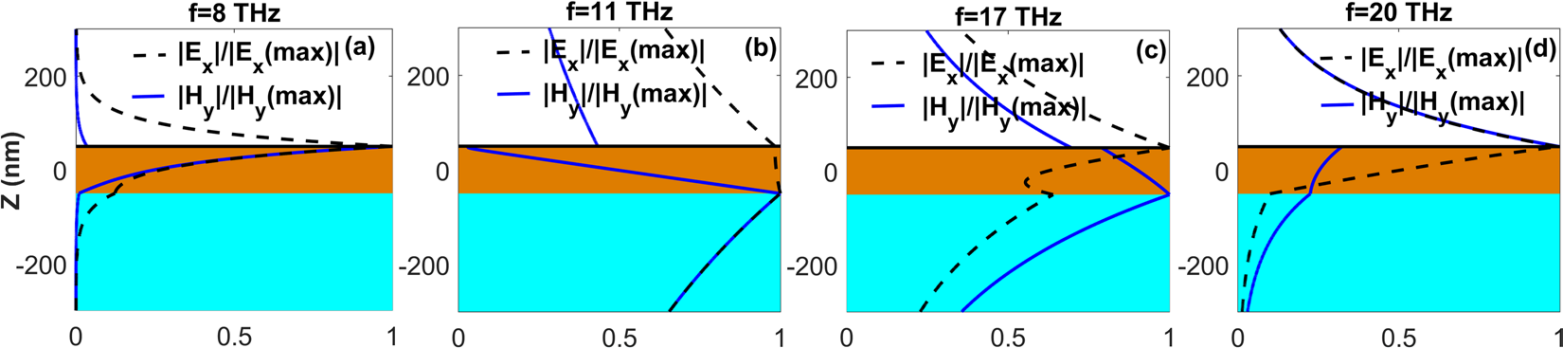
Profiles of the normalized |*E*_*x*_| (dashed black line) and |*H*_*y*_| (solid blue line) for SPPPs of the air/graphene/LiF/glass waveguide with *t* = 100 *nm*. (**a**) *f* = 8 *THz*, (**b**) *f* = 11 *THz*, (**c**) *f* = 18 *THz*, and (**d**) *f* = 20 *THz*. Glass substrate, LiF film, and air regions are highlighted in aqua, light brown and white, respectively. The graphene layer is schematically shown by solid black line at *z* = *t*/2.

**Figure 7:**
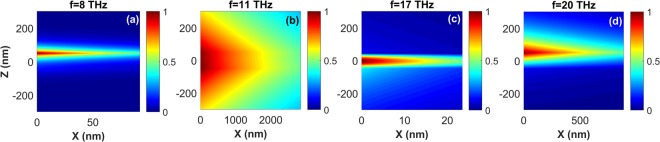
Spatial distribution of the normalized |*E*_*x*_| for the SPPPs supported by the air/graphene/LiF/glass waveguide with *t* = 100 *nm*; (**a**) *f* = 8 *THz*, (**b**) *f* = 11 *THz*, (**c**) *f* = 18 *THz*, and (**d**) *f* = 20 *THz*.

**Figure 8:**
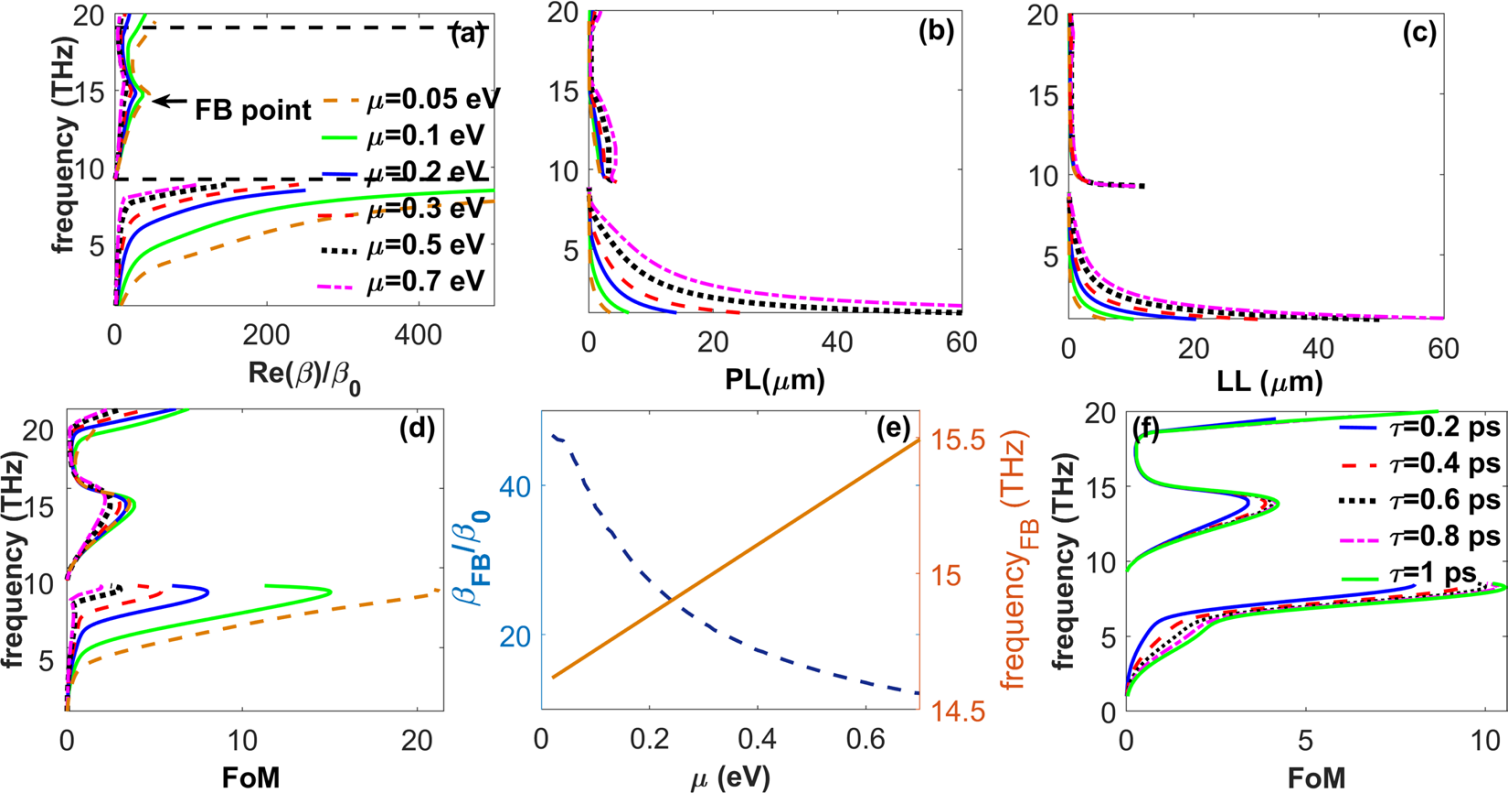
(**a**) Dispersion, (**b**) propagation length, (**c**) localization length, (**d**) figure of merit of the SPPPs supported by air/graphene/LiF/glass waveguide with *t* = 100 *nm* for *μ* = 0.05 *eV* (dashed brown lines), 0.1 *eV* (solid green lines), 0.2 *eV* (solid blue lines), 0.3 *eV* (dashed red lines), 0.5 *eV* (dotted black lines), and 0.7 *eV* (dashed pink lines). The FB point in panel (a) indicates the frequency and wavenumber, at which the forwardly propagating modes are turned to propagate backwardly and vice versa. Panel (**e**) illustrates the changes in the frequency and wavenumber at the FB point via variations in *μ*. (**f**) *FoM* of the guided modes supported by the case of graphene/100 *nm* LiF/glass for different values of *τ*.

For a deeper insight into the features of the SPPPs, in Fig. 6 we present field profiles of the SPPP modes for the case of *t* = 100 *nm*, at four different frequencies. In agreement with Fig. 5(c), the mode profiles at *f* = 11 *THz* [Fig. 6(b)] and *f* = 18 *THz* [Fig. 6(c)] are very similar to the ones illustrated in Fig. S5(c,d). This similarity verifies that at these frequencies, which are inside the polaritonic gap of LiF, the SPPPs possess mostly SPhPs characteristics. At the same time, for the frequencies outside the polaritonic gap, i.e., at *f* = 8 *THz* [Fig. 6(a)] and *f* = 18 *THz* [Fig. 6(d)], the mode profiles resemble those shown in Fig. S2(a,d). In tnis case, SPPPs attain characteristics of graphene SPPs. In order to verify the SPPPs propagation length for the case of *t* = 100 *nm* in Fig. 5(b), two-dimensional field distributions of the SPPPs are also illustrated in Fig. 7. As expected, at *f* > *f*_*TO*_ propagation in the waveguide with noticeably small confinement is possible, as shown in Fig. 7(b–d). On the other hand, Fig. 7(a) confirms that the slowly propagating SPPPs at *f* < *f*_*TO*_ are strongly confined inside the waveguide and show high *FoM*. As mentioned above, tunability of the optical properties via changing *μ* can be considered as the most advantageous characteristic of graphene over other plasmonic and phononic materials. Therefore, we finally investigate the effect of changes in *μ* on the characteristics of SPPPs supported by the air/graphene/LiF/glass waveguide with *t* = 100 *nm*. The results in Fig. 8(a–e) show that by changing *μ*, dispersion, propagation and localization characteristics of the SPPPs can be efficiently tuned at frequencies inside and outside of the RS band of LiF. In Fig. 8(d) it is illustrated that the maximal *FoM* of the forwardly propagating slow SPPPs at *f* < *f*_*TO*_ can be significantly increased by decreasing *μ*, that is associated with decrease of *v*_*g*_ and increase of wavenumber. Moreover, it is possible to tune the frequency of the forward-to-backward (FB) wave transition at *f*_*TO*_ < *f* < *f*_*LO*_. In Fig. 8(a) the transition point is indicated as FB point. Figure 8(e) provides more evidence of the frequency and wavenumber tuning for the FB point. By decreasing *μ*, it can be shifted toward lower frequencies and larger wavenumbers. From a practical point of view, the presence of substrate may add some additional effects, such as electron impurity and electron losses, on the electronic and finally optical properties of graphene. These effects can be included in the calculations by the phenomenological relaxation time (*τ*) or the electron mobility as $e{v}_{F}^{2}\tau /\mu$ where ${v}_{F}=c/\mathrm{300}$. Therefore, in Fig. 8(f) we investigated how different values of *τ* can affect *FoM* of the hybrid guided modes supported by the graphene/LiF/glass waveguide for *t* = 100 *nm*. As it is observed from Fig. 8(f), a 5-time increase in the relaxation time leads to almost 1.3-time enhancement of the *FoM* of the modes. More investigations reveal that a reverse trend can be observed by decreasing *τ*. Moreover, for frequencies larger than the optical phonon frequency (*f* > 48.4 *THz*), electron-phonon scattering can considerably affect *τ*^20^. The inter-layer coupling is also another mechanism that can considerably amend the relaxation time^68,69^. One more important point should be highlighted here is that, in general, nonlocal optical conductivity of graphene can be considered in the calculations^16–20^. As explained in^16–20^, once the substrate of graphene is low-index and not strongly dispersive, taking the local optical conductivity of graphene in the calculations leads to a precise description of the modal characteristics of the guided waves for low frequencies and small values of the wavenumbers; i.e. for *β*/*β*_0_ < 500 and *f* < 20 *THz*. To the present, most of the studies on the investigation of plasmon-phonon hybridizations in the graphene/polar heterostructures have been done in the local regime^28,40–52^. However, in order to gain more practical insight into the hybridization mechanism and the modal characteristics of the guided waves supported by those systems, considering the nonlocal effects in the calculations will be the subject of our future studies.

## Conclusion

To summarize, we have theoretically studied two types of the structures with graphene and one auxiliary structure with LiF, a polar dielectric, which all may support surface waves in the far-IR region. Graphene is a well-known plasmonic material capable of supporting low-loss tunable SPPs in the THz and IR regions. On the other hand, LiF is a strongly dispersive phononic material which can support SPhPs within its Reststrahlen band (polaritonic gap), i.e., at 9.22 *THz* < *f* < 19.1 *THz*. Therefore, combining graphene and LiF components in one structure promises surface waves with the improved and unusual characteristics at the far-IR region. Moreover, from the investigation of graphene SPPs for different dispersion-free dielectric substrates, it has been understood that the use of lossless high-permittivity substrates allows us to considerably increase *FoM* of the structure. This effect is coherent with the slowing of surface plasmons due to increase of substrate permittivity. From the investigation of SPhPs supported by thin films of LiF on different substrates, it has been concluded that a 10 *nm*-thick film of LiF on glass substrate is a distinguishable candidate for obtaining highly confined and largely propagating SPhPs within 10–18 *THz*, when tunability is not required. Finally, we studied the effects that appear due to combining graphene with a thin film of LiF and a glass substrate in one structure, so that the effects of wide-range permittivity of LiF and tunability of graphene co-exist. We have shown that due to the hybridization of graphene SPPs and LiF SPhPs, SPPPs are supported by the graphene-LiF waveguide, which may show very different behavior within the neighboring frequency regions, i.e., below, inside, and above the polaritonic gap. This characteristic makes the graphene-LiF heterostructures beneficial for multifunctional applications. In particular, (i) due to the large values of permittivity of LiF just below the transverse optical phonon frequency, tunable (by variations in chemical potential), forwardly propagating, slow SPPPs with 3.2 times larger *FoM* than graphene SPPs are supported within 5–9 *THz*, when the thickness of the LiF layer is 100 *nm*; (ii) the coherence between the extent of wave slowing and the increase of *FoM* takes place; (iii) backward SPPPs with tunable propagation and localization characteristics can be supported. These features make graphene/LiF/glass heterostructures versatile candidates for waveguides, sensors and absorbers in the far-IR region. While LiF has been chosen owing to a wide polaritonic gap and relatively low losses at the far-IR, the potential of other polar dielectrics, e.g., GaAs, InP, and CaF_2_ in obtaining tunable hybrid surface waves with improved characteristics may be a subject of future studies. The obtained results can be viewed from the perspective of new advanced physical scenarios and applications for graphene, but simultaneously from the perspective of those ones for polar dielectrics. They indicate that radiation and manipulation of THz waves at the far-IR by using the hybridized SPPPs in graphene-polar dielectric heterostructures is a promising research direction.

## Methods

We first obtained the appropriate boundary conditions for TM polarization, then using a standard direct-matching technique^25,67^ the dispersion relations of the guided modes supported by the aforementioned three structures were derived. Considering real values of *ω* and complex values of *β* in the calculations, dispersions, propagation and localization characteristics of the guided modes were extracted using custom-made MATLAB codes. The 1D and 2D mode profiles presented in the manuscript were also extracted by custom-made MATLAB codes and verified by 3D Finite Difference Time Domain (FDTD) calculations^70^. Taking the effective mode index obtained from the MATLAB codes, we performed the FDTD simulations using a mode source with the periodic boundary condition in the plane of graphene and applying perfectly matched layer boundary condition at the out of plane boundaries. Moreover, the graphene and LiF layers were, respectively, modeled by Eqs. (3) and (4) in the calculations.

## Electronic supplementary material

Supplementary Information
