# Supplementary material for: Tailoring far-infrared surface plasmon polaritons of a single-layer graphene using plasmon-phonon hybridization in graphene-LiF heterostructures
Source: Sci Rep. 2018 Sep 4;8:13209. doi: 10.1038/s41598-018-31049-6 (PMC6123435; doi:10.1038/s41598-018-31049-6)
Supplement: Supplementary file 1 — Supplementary Information [file 41598_2018_31049_MOESM1_ESM.pdf]

# Tailoring far-infrared surface plasmon polaritons of a single-layer graphene using plasmon-phonon hybridization in graphene-LiF heterostructures

**Hodjat Hajian[1*], Andriy E. Serebryannikov[2,3], Amir Ghobadi[1,4], Yigit Demirag[1], Bayram Butun[1], Guy A. E. Vandenbosch[2], and Ekmel Ozbay[1,4,5,**]**

[1]Nanotechnology Research Center, Bilkent University, 06800 Ankara, Turkey

[2]ESAT-TELEMIC, Katholieke Universiteit Leuven, B-3000 Leuven, Belgium

[3]Faculty of Physics, Adam Mickiewicz University, 61-614 Poznan, Poland

[4]Department of Electrical and Electronics Engineering, Bilkent University, 06800 Ankara, Turkey

[5]Department of Physics and UNAM-Institute of Materials Science and Nanotechnology, Bilkent University, 06800 Ankara, Turkey

[*]hodjat.hajian@bilkent.edu.tr, [**]ozbay@bilkent.edu.tr

## 1. Tunable characteristics of surface plasmon polaritons of a single-layer graphene

It is known that one of the most impactful properties of the SPPs supported by graphene is their tunability by varying $\mu$. In Fig. S1, by taking $\varepsilon_s = 35$, we examine the SPP characteristics.

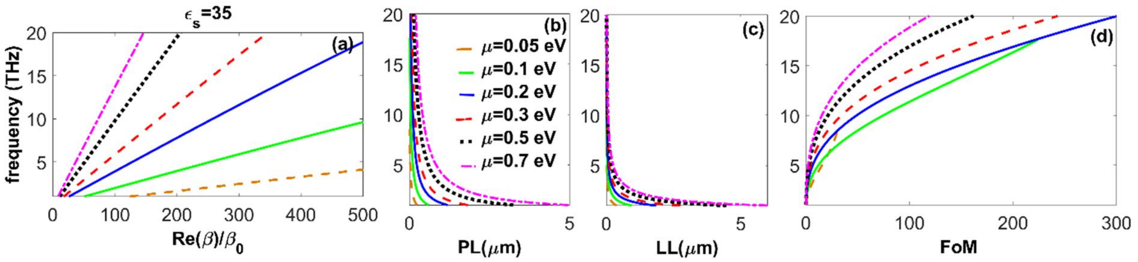


Fig. S1. (a) Dispersion, (b) propagation length, (c) localization length, and (d) figure of merit of the SPPs supported by a single-layer of graphene on a lossless substrate with $\varepsilon_s = 35$).The chemical potential is 0.05 eV (dashed brown line), 0.1 eV (solid green line), 0.2 eV (solid blue line), 0.3 eV(dashed red line), 0.5 eV (dotted black line), and 0.7 eV (dashed-dotted pink line).

Figure S1(a) shows that by decreasing $\mu$, SPPs are supported within a narrower range of frequency and for larger values of $\beta'$; i.e., for the smaller values of $\lambda_{sp}$ and $v_g$. By varying $\varepsilon_s$, one can change the wavenumber range attainable by the same variations in $\mu$ (not shown). It is observed in Fig. S1(b) and Fig. S1(c), respectively, that the decrease in $\mu$ leads to a reduction in SPPs' PL, while swelling the modes' confinement. These behaviors, in total, cause a rise in the $FoM$ of the graphene SPPs, see Fig. S1(d). For the operating frequencies from 7 THz to 17 THz, $\mu = 0.1$ eV yields SPPs with the largest $FoM$, while at $f > 17$ THz,

$\mu = 0.2$ eV produces such optimal SPPs. Moreover, when $f < 7$ THz, it occurs at $\mu = 0.05$ eV. Qualitatively, the effect of increase of $\mu$ is similar to the effect of decrease of $\varepsilon_s$ for $\lambda_{sp}$, but the same cannot be said regarding FoM, for which an optimal value of $\varepsilon_s$ can exist. For a richer insight onto the localization of the graphene SPPs, field profiles are also presented in Fig. S2 at four different frequencies. For convenience of demonstration, we take here $\varepsilon_s = 2.25$, while the behavior for $\varepsilon_s = 35$ is similar but shows stronger mode confinement, as predicted by the results in Fig. S1(c).

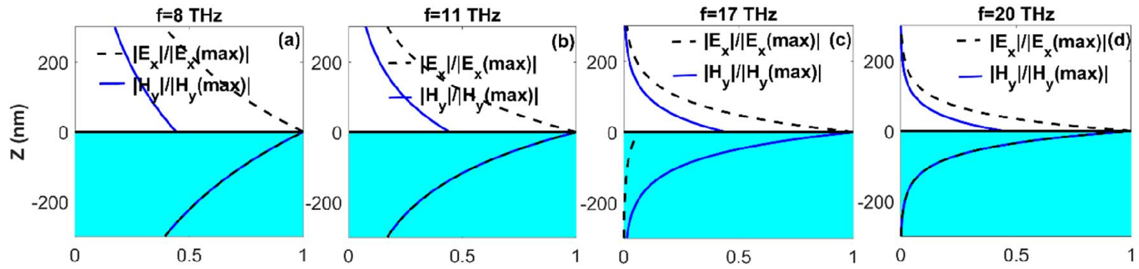


Fig. S2 Profiles of the normalized $|E_x|$ (dashed black line) and $|H_y|$ (solid blue line) of the SPPs supported by the air/graphene/glass structure for $\mu = 0.2$ eV(permittivity of glass is $\varepsilon_s = 2.25$). The solid black horizontal line shows location of the graphene layer. The substrate and air regions are highlighted in aqua and white, respectively.

## 2. Effect of different substrate and thicknesses on the SPhPs of thin LiF films

Here we investigate the effect of $\varepsilon_s$ on the modal characteristics of the LiF waveguide with $t = 10\ nm$. The results are presented in Fig. S3.

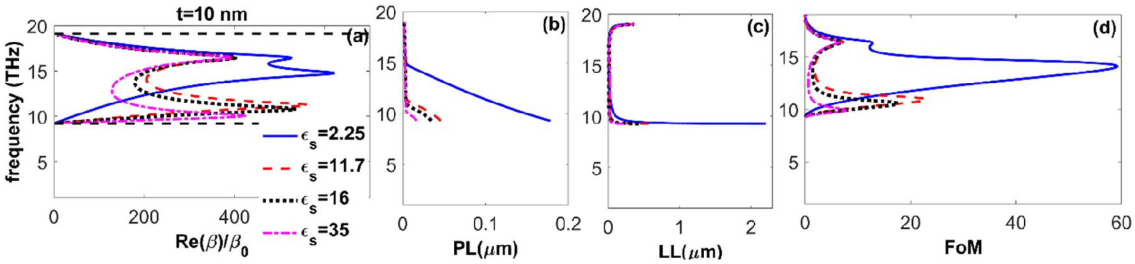


Fig. S3 (a) Dispersion, (b) propagation length, (c) localization length, and (d) figure of merit of the SPhPs supported by air/LiF waveguides at $t = 10$ nm, for substrates with $\varepsilon_s = 2.25$ (solid blue line), $\varepsilon_s = 11.7$ (dashed red line), $\varepsilon_s = 16$ (dotted black line), and $\varepsilon_s = 35$ (dashed-dotted pink line). Dashed black lines in panel (a) show the edges of the polaritonic gap of LiF.

Figure S3(a) shows that the increase of $\varepsilon_s$ considerably affects dispersion of the SPhPs, so that the maximal wavenumber is downshifted and $\lambda_{sp}$ is increased. This increase in $\lambda_{sp}$ and the reduction in the PL [see Fig. S3(b)] lead to a noticeable swelling in the modal losses and, finally, in the drop of $FoM$ of the SPhPs modes. As observed in Fig. S3(c), increase of $\varepsilon_s$ makes the modes more localized, but this positive effect cannot compensate the negative effect of the losses on the $FoM$ of the waveguide. Note that the value of $\varepsilon_s$, which provides the largest wavenumber, can be different for different frequencies, e.g., compare vicinities

of 10 and 15 THz in Fig. S3(a). Figure S4 illustrates $FoM$ of the SPhPs for different values of $t$, and $\varepsilon_s$. As expected from the results of Figs. 3 and S3, by increasing $\varepsilon_s$ and $t$, $FoM$ is considerably reduced. Thus, as far as we are interested in a phononic waveguide with the highest FoM within 10-18 THz, the structure with $\varepsilon_s = 2.25$ and $t = 10\ \mathrm{nm}$ can be considered as the optimal design. Next, we investigate mode profiles of the LiF phononic waveguide for t=10 nm and t=100 nm.

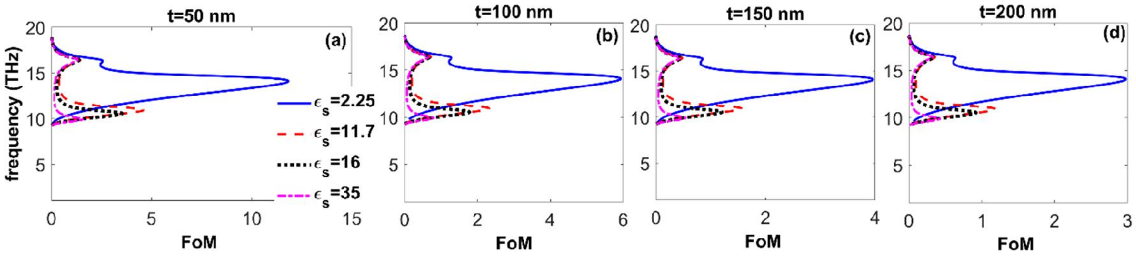


Fig. S4 Figure of merit of the SPhPs supported by films of LiF with different thicknesses on different substrates: (a) $t = 50\ \mathrm{nm}$, (b) $t = 100\ \mathrm{nm}$, (c) $t = 150\ \mathrm{nm}$, and (d) $t = 200\ \mathrm{nm}$; for each panel, $\varepsilon_s = 2.25$ (solid blue line), $\varepsilon_s = 11.7$ (dashed red line), $\varepsilon_s = 16$ (dotted black line), and $\varepsilon_s = 35$ (dashed-dotted pink line).

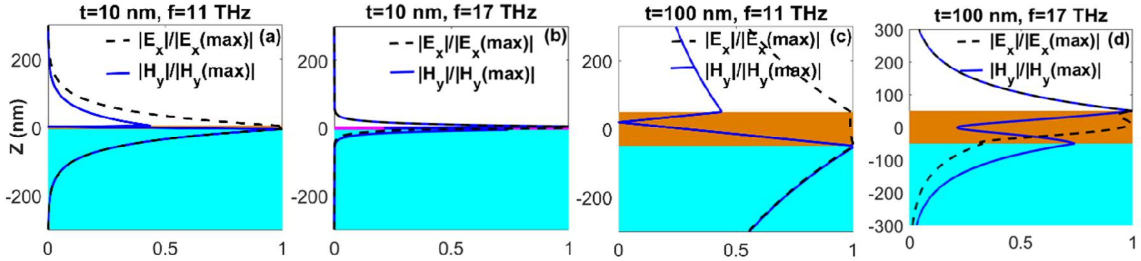


Fig. S5 Profiles of the normalized $|E_x|$ (dashed black lines) and $|H_y|$ (solid blue lines) of the SPhPs supported by the air/LiF/glass ($\varepsilon_s = 2.25$) waveguides at $t = 10\ \mathrm{nm}$ [panels (a) and (b)] and $t = 100\ \mathrm{nm}$ [panels (c) and (d)]; $f = 11\ \mathrm{THz}$for panels (a) and (c) and $f = 18\ \mathrm{THz}$ for panels (b) and (d). Substrate (lower), LiF film (middle), and air (upper) regions in the mode profiles are highlighted in aqua, light brown and white, respectively.

Figures S5(a) and S5(b) present the mode profiles of the SPhPs supported at $t = 10\ \mathrm{nm}$ when $f = 11\ \mathrm{THz}$ and $f = 18\ \mathrm{THz}$, respectively. One can see that the mode confinement increases for higher frequencies. Moreover, by comparing these results with Fig. S2, it is understood that the SPhPs of the 10 nm-thick LiF waveguide are even stronger confined than the graphene SPPs in 10-18 THz. This considerable confinement, which is in agreement with the LL results shown by solid blue curve in Fig. 3(c), is the main cause of the large values of $FoM$ of this waveguide. The mode profiles for the case of $t = 100\ \mathrm{nm}$ are shown in Figs. S5(c) and S5(d) for comparison.
